# Supplementary material for: A risk scoring model of COVID-19 at hospital admission
Source: PLoS One. 2023 Jul 20;18(7):e0288460. doi: 10.1371/journal.pone.0288460 (PMC10358923; doi:10.1371/journal.pone.0288460)
Supplement: Attachment — Submitted filename: Response to Reviewers.docx [file pone.0288460.s001.docx]

***1. The manuscript is not scientifically organized, and the tables and figures are not presented in an academic style that is suitable for publication.***

The article is organized in the standard way for many scientific articles: Introduction, Methods, Results, Discussion and Conclusions.

Tables and figures were attached according to the guidelines suggested by PlosOne. The tables were incorporated in the text in the simplest possible way so that they could be adapted by the editor if necessary and the figures were attached in separate files as the submission manual suggests. In the new proposed manuscript, we altered 3 tables that were in the format of an R output image.

***2. Lack of details on the setting and conduction of study and poor interpretation of results.***

This study was conducted from a database of over 50,000 people, of which over 12,000 individuals have died.

It contains the following information: Date of birth, date of entry and exit from the hospital, sex, health unit where the patient was admitted (which allowed us to, based on the information from the Portuguese Ministry of Health regarding hospital capacity, infer the pressure in terms of average hospital capacity per hospitalization and use this information in the model) and the various comorbidities associated with each patient (through a sophisticated process it was also possible to assign a risk score to each patient) – all this is described in detail in the manuscript.

Based on this material, we built a regression model that allows us to estimate the probability of death for each patient diagnosed with COVID and associate this probability to its respective confidence interval. In addition to that, we built profiles for the most frequent patterns with an associated probability of death (survival) to each, allowing us to have an idea of the increase in risk for a given factor. Furthermore, we retrospectively simulated some of these profiles to understand the model's ability to discriminate between survivals and deaths. It was also possible to objectively quantify the importance of age in terms of COVID mortality - about 47% compared to the 5 factors available.

We hope to have been able to enlighten the importance of this study and the results obtained, specially for an in-depth analysis of the strategy that was followed by most countries during the pandemic outburst.
