# Supplementary material for: A risk scoring model of COVID-19 at hospital admission
Source: PLoS One. 2023 Jul 20;18(7):e0288460. doi: 10.1371/journal.pone.0288460 (PMC10358923; doi:10.1371/journal.pone.0288460)
Supplement: Attachment — Submitted filename: Response to Reviewers.docx [file pone.0288460.s002.docx]

Journal Requirements

# 1. “Please ensure that your manuscript meets PLOS ONE's style requirements, including those for file naming. The PLOS ONE style templates can be found at link and link2”

Manuscript has been carefully revised and now meets PLOS ONE’S style requirements.

# 2. “We note that you have stated that you will provide repository information for your data at acceptance. Should your manuscript be accepted for publication, we will hold it until you provide the relevant accession numbers or DOIs necessary to access your data. If you wish to make changes to your Data Availability statement, please describe these changes in your cover letter and we will update your Data Availability statement to reflect the information you provide”

# 3. “Please note that PLOS ONE has specific guidelines on code sharing for submissions in which author-generated code underpins the findings in the manuscript. In these cases, all author-generated code must be made available without restrictions upon publication of the work. Please review our guidelines at link and ensure that your code is shared in a way that follows best practice and facilitates reproducibility and reuse

We are ready to provide you with both the data and the code used to build the logistic model, upon acceptance of our paper.

# 4. “Your abstract cannot contain citations. Please remove any existing citations from the abstract section. You may only include citations in the body text of the manuscript, and please ensure that they remain in ascending numerical order on first mention.”

As suggested, the abstract now has no citations.

Review Comments

# 1. “I would suggest the authors look for some professional English editorial help to avoid grammar errors and make the manuscript fluent and easier to read”

The writing has been carefully revised as suggested by reviewer.

# 2. “Abstract: the Conclusion part is missing in Abstract”

Conclusions were added to the Abstract section of the paper.

# 3. “The way the authors created the diagnoses score is unclear. More details (or specific example how the scores are created) are needed.”

The approach applied followed a scorecard model approach, which is meant to be simpler to explain and apply. Two of the most common cited references in the literature are, for instance: [Thomas, Edelman, Crook; Credit Scoring and Its Applications Society for Industrial and Applied Mathematics (1983)] [Anderson, R.; Credit Scoring Toolkit - Theory and Practice for Retail Credit Risk Management and Decision Automation, Oxford University Press (2007)].

Further explanation on this topic was added to the manuscript in the Methods section.

# 4. “The context in the manuscript is too messy. Please refer to the published paper (from PLOS ONE or any other academic journals) to clean up the Methods and Results section. Specifically:”

## a) “Row 76 to 79: it will be good to have a data flowchart to reflect the subject number change by inclusion or exclusion creation”

A flowchart was added to the manuscript, explaining the transformations made to the initial database.

## b) “Row 90-99: it’s unnecessary to show the variable name from original dataset”

The names presented correspond to the variable names used in our model and a brief description clarifying what each variable means. However, presentation has been simplified.

## c) “Row 101: Table 1 are unnecessary and can be moved to supplementary tables if the authors insist to keep them.”

Table 1 was removed according to suggestion.

## d) “Row 108: Table 2, there are too many facilities and it would better to use a bar plot to show the number of patients and death rate.”

Information previously presented in Table 2 was converted to a bar plot (Fig. 2), per suggestion.

## e) “Table 3, 4 and 5 can be combined as one descriptive table and should be placed in Results section.”

The suggested changes were made – Tables 3,4 and 5 were combined into one single table (Table 1) and were moved to the Results section.

## f) “Row 125: Table 6 can be merged into Table 2 and presented as plot as well”

Tables 2 and 6 have been combined as suggested as were converted into a bar plot (Fig. 3).

## g) “Row 152: Table 7 isn’t presented in academic style. ORs with CIs are better”

## h) “Why the presented results content are different in Table 8 (Df/Deviance/Resid.Df…) and Table 9 (Estimate/Std./Error)? These results are not presented in standard way. No one should directly present formula and output results from analytical software. Please refer to the published paper to make it more rigorous.”

Tables 7 and 8 have been combined (Table 2) and the results of the univariate analysis are now presented in a standard way, per suggestion.

## i) “Row 169: could you cite reference to use “percentage by dividing each coefficient by the sum of all coefficients” to describe the variable importance?”

Further explanation as to why this approach was followed was added to the manuscript, as well as reference (Multiple Regression section in Results), as suggested.

## j) “Row 179: Table 11 needs to be revised like Table 9, and the interpretation needs improvement.”

Tables 9 and 11 were revised and the results are now presented in a standard way (they now correspond to Tables 3 and 5, respectively). Interpretation of the results has also been improved.

## k) “ Row 223: Figure 5 and Figure 6 can be merged as one plot to show the difference of the boundary line between male and female.”

Figures 5 and 6 have been merged into one single plot (Fig. 8)

## l) “Row 242: Table 12 can be transformed as a plot using Age as x-axis and probability as y-axis. Then it will be easier to compare among different profile and gender groups.”

Table 12 has been transformed into a plot, per suggestion.
